# The Relationship between Body Composition and Physical Fitness and the Effect of Exercise According to the Level of Childhood Obesity Using the MGPA Model

**DOI:** 10.3390/ijerph19010487

**Published:** 2022-01-02

**Authors:** Chae Kwan Lee, Young Kyun Sim, Jae-Hoon Lee, Jang Soo Yook, Soo-Min Ha, Eun Chul Seo, Wi-Young So, Hyun Ryun Kim, Woo-Min Jeong, Bong Oh Goo, Jin-Wook Chung, Min-Seong Ha

**Affiliations:** 1 Department of Physical Therapy, Catholic University of Pusan, 57 Oryundae-ro, Geumjeong-gu, Busan 46252, Korea; cklegend@naver.com (C.K.L.); kbo905@cup.ac.kr (B.O.G.); 2 Department of International Sports, Dankook University, 119 Dandae-ro, Dongnam-gu, Cheonan-si 31116, Korea; simco76@dankook.ac.kr; 3 Department of Sports Science, University of Seoul, 163 Seoulsiripdae-ro, Dongdaemun-gu, Seoul 02504, Korea; leejh0923@gmail.com; 4 Center for Functional Connectomics, Brain Research Institute, Korea Institute of Science and Technology (KIST), 5 Hwarang-ro 14-gil, Seongbuk-gu, Seoul 02792, Korea; soulyook84@gmail.com; 5 Laboratory of Exercise Physiology, Department of Physical Education, Pusan National University, 2 Busandaehak-ro 63beon-gil, Geumjeong-gu, Busan 46241, Korea; fantasista@pusan.ac.kr; 6 Department of Physical Education, Wonkwang University, 460 Iksan-daro, Iksan 54538, Korea; eunchulseo17@wku.ac.kr; 7 Sports Medicine Major, College of Humanities and Arts, Korea National University of Transportation, Chungju 27469, Korea; wowso@ut.ac.kr; 8 Department of Physical Education, Woosuk University, 443 Samnye-ro, Samnye-eup, Wanju-gun 55338, Korea; khr0615@naver.com; 9 WellCare Korea Co., Ltd., 26 Wadong-ro, Danwon-gu, Ansan-si 15265, Korea; wellcarek@naver.com; 10 Department of Sports Culture, College of the Arts, Dongguk University, 30 Pildong-ro 1-gil, Jung-gu, Seoul 04620, Korea; cjw826@dongguk.edu

**Keywords:** body composition, physical fitness, childhood obesity, exercise MGPA analysis

## Abstract

Childhood obesity can lead to adulthood obesity with adverse effects. Since body composition and physical fitness differ depending on the obesity degree, a systemic analysis could help classify that degree. We used three study designs based on the obesity degree (body mass index [BMI] as a reference) for our objectives. First, we identified the relationship between body composition and physical fitness. Second, we determined the effects of exercise on body composition and physical fitness. Third, we performed a path analysis of the impact of exercise on body composition and physical fitness, and verified those effects among the groups. In study 1, 164 10-year-old subjects were divided into four groups: 33 in the normal weight (NO), 34 in overweight (OV), 54 in obesity (OB), and 43 in the severe obesity (SOB) group. In study 2, 101 participants from study 1 who wished to participate in the exercise program were divided into four groups (same criteria). The exercise program (three times a week for 60 min, for 16 weeks) consisted of sports and reinforcement exercises of increasing intensity. Body composition was measured by body weight, percentage of body fat (%BF), muscle mass, skeletal muscle mass (SMM), and body mass index (BMI). In contrast, physical fitness was measured by muscular strength, flexibility, muscular endurance, agility, and balance. As a result, all body composition variables were higher in the SOB group than in the other groups. Physical fitness, muscular strength and balance, and agility were highest in the SOB, NO, and OV groups, respectively. Pearson’s correlation revealed that muscular strength was associated with height and body weight across all groups. Agility showed a negative correlation with %BF in the NO, OB, and SOB groups. SMM was positively correlated in the OB and SOB groups. After the exercise intervention, BMI and the %BF of the SOB group were significantly reduced (*p* < 0.01, and *p* < 0.001, respectively), while SMM presented a significant increase (*p* < 0.001). Height also showed a significant increase in all groups (*p* < 0.001). Among physical fitness variables, muscular strength, flexibility, muscular endurance, and balance showed a significant increase in all groups, while a significant increase in power was observed in only the OB and SOB groups. As for the effects of the body composition on physical fitness after exercise intervention, the greatest impact was observed for balance, muscular strength and agility, and muscular endurance in NO, OV, and OB groups, respectively. In conclusion, the body composition, physical fitness relationship, and the effects of exercise intervention on them differed depending on the obesity degree. Furthermore, the results varied according to the obesity degree. Thus, our study highlights the importance of creating particular exercise programs for the effective prevention and treatment of childhood obesity considering the obesity degree.

## 1. Introduction

Childhood obesity is a major public health issue; 50% of cases lead to obesity in adolescence, 80% of which lead to obesity in adulthood [1]. Hence, it is important to prevent and diagnose obesity in childhood to be able to address the high risk for chronic diseases in adulthood [2,3].

Body mass index (BMI) is a reliable tool for assessing obesity in children and adolescents, given the limitations of other measurement tools used to measure the body in clinical practice [4]. Though errors in BMI are, at a young age, due to rapid physical development, BMI data and growth charts by age are reliable in diagnosing obesity [5]. Since weight gain appears as a J-curve or U-curve in relation to mortality, mortality rates are not only directly related to BMI [6] but are an important predictor of cardiovascular disease [7]. Therefore, we classified obesity by degree based on BMI for the purpose of this study.

Among the causes of childhood obesity are environmental factors, genetic factors, endocrine disorders, and sleep [8]. Physical activity in childhood tends to decrease by 6.9% every year as age increases [9], thus contributing to obesity. However, the coronavirus disease (COVID-19) pandemic and the control measures introduced have resulted in approximately a 37% decrease in physical activity [10]. A stepwise method for resolving childhood obesity initially is a change of diet and exercise regimen [4,8] by engaging in physical activity for a minimum of 60 min, three times a week [11]. Physical activity also enhances the overall health and well-being of the child, improves the body composition, including (body fat and muscle), cardiopulmonary function, muscular strength, muscular endurance, and flexibility [12].

A cross-sectional study was conducted with 423 children aged 8–10 years. It compared physically active and inactive children. It was found that children who played ball games (soccer, basketball, volleyball), badminton, and gymnastics, presented better body composition and physical fitness than those who did not play any sports [13]. The study of Alberga et al. (2016) [14] on 304 adolescents found that aerobic exercise showed the greatest impact in cardiopulmonary training, while participants engaged in resistance and combination exercises showed muscular strength and endurance improvement. Hence, many researchers recommend combination exercise as a more effective option for the overall physical health of growing children [15].

Most previous research has focused on identifying the relationship between body composition and physical fitness, or examining the intervention effects of exercise in obese and normal children. However, when exploring the relationship between body composition and physical fitness in 225 adolescents, significant differences were found between the overweight and obese groups and the normal-weight group in the long jump, agility, and cardiorespiratory fitness tests [16]. Although research should also be conducted according to the degree of obesity, studies examining the differences in body composition and physical fitness, or the effects of intervention by classifying the degree of obesity, are still lacking. In order to classify by degree of obesity and analyze variables, a method of simultaneous analysis of the causal relationship between constituent concepts is necessary [17]. Structural equation modeling (SEM) is widely used in non-experimental studies [18]. Still, no experimental studies have used a multi-group path model to investigate the relationship between body composition and physical fitness for childhood obesity. The analysis of the MGPA could serve as a strength for clearly explaining the purpose of our study.

This study aimed to examine the relationship between body composition and physical fitness by obesity degree, to determine the effect of exercise on body composition and physical fitness, and to analyze the obesity degree of different groups (classified by BMI), while applying a multi-group path analysis (MGPA) model. We hypothesized that the severe obesity (SOB) group would have worse health in terms of body composition and physical fitness than the other groups. After a 16-week exercise intervention, SOB would show the greatest change in body composition and physical fitness, and the MGPA model will explain the effect of exercise on body composition and physical fitness in participants with childhood obesity.

## 2. Materials and Methods

### 2.1. Study 1. Analysis of the Relationship between Body Composition and Physical Fitness in Obese Children (Correlation, Stepwise Multiple Regression Analysis)

#### 2.1.1. Design, Participants

Study 1 was a descriptive correlation study aiming to investigate the body composition and physical fitness relationship in children with obesity, and identify variables affecting physical fitness. A total of 164 10-year-old children living in Korea (107 boys, 57 girls) were classified into 4 groups according to the degree of obesity (normal weight (NO); *n* = 33, overweight (OV); *n* = 34, obesity (OB); *n* = 54, and severe obesity (SOB); *n* = 43). The parents of the children willing to participate were given a detailed explanation of the objectives and intentions of the study, and provided written consent before enrolling their children. The exclusion criteria were having received drug therapy in the past six months, having engaged in regular exercise, or having had previous musculoskeletal disease. During the study period, the use of medications that could affect the experiment was restricted.

#### 2.1.2. Measures

Participants were required to maintain a minimum of 8 h of fasting, with no vitamin intake or vigorous exercise. Body composition was determined using Inbody J10 (Biospace Corp., Seoul, Korea), evaluating body weight (kg), percentage of body fat (%BF; %), muscle mass (kg), and skeletal muscle mass (SMM; kg). The formula for BMI used was weight in kilograms divided by height in meters squared. For physical fitness, muscular strength (kg) was measured using the grip dynamometer (TKK-5403, Takei Corp., Niigata, Japan, kg); flexibility (cm) was measured using the sit-and-reach meter (TKK-5404, Takei Corp., Japan, cm); muscular endurance (times) and agility (cm) were measured by performing sit-ups and long jumps in place, respectively. Balance (s) was measured by standing on one leg with eyes closed.

##### Flexibility: Sit-and-Reach (Centimeters; cm)

Participants sit and bend forward (shoes off) so that the entire soles of both feet are in contact with the sit-and-reach meter, with knees straightened and both hands extended to the measuring instrument as much as possible. Two measurements are taken, and the best score is recorded to 0.1 cm.

##### Muscular Strength: Grip Strength (Kilograms; kg)

Participants stand in a comfortable position with arms straight on either side of the torso at 15° while applying force. During the measurement, the dynamometer is not in contact with the body, and the relative grip strength is recorded in units of 0.1 kg.

##### Muscular Endurance: Sit-Ups (Times)

Participants lie on a mat with feet 30 cm apart, the knees bent at 90°, and both hands behind the head. They sit up by moving the upper body up to touch the knees with the elbows on both sides, and then lie back to the original position. This movement is maxed out in 60 s.

##### Agility: Long Jump in Place (Centimeters; cm)

Participants stand in front of a white line. They jump as far as they can. The position of the heels touching the ground is marked. The measurement is taken twice, and the best score is used.

##### Balance: Standing on One Leg with Eyes Closed (Seconds; s)

Participants are allowed to choose the most comfortable leg to stand on, with the opposite leg bent up while placing hands on the waist and closing eyes. Time is measured while they stand on one leg. To prevent injuries, the researcher assists in a position not interfering with the measurement.

#### 2.1.3. Statistical Analyses

The required sample size was calculated using the G-power version 3.1 Window program (Kiel University, Kiel, Germany), based on a 0.25-point effect size (default), an alpha level of 0.05, and 70% power. The mean and standard deviation were calculated using SPSS 23.0 (IBM Corp., Chicago, IL, USA). Pearson’s correlation was used to examine the relationship between body composition (BMI, height, weight, %BF, SMM) and physical fitness (strength, flexibility, muscular endurance, agility, and balance) (Tables S1 and S2). Among the factors showing significant correlation, strength, muscular endurance, agility, and balance were used as dependent variables, while body composition was used as the independent variable to conduct stepwise multiple regression analysis to establish the relationship between body composition and physical fitness (Table S3). After using one-way ANOVA to analyze the differences in the body composition and physical fitness in children by obesity degree, a post-hoc test was conducted using Scheffe when the sample size was different, but homogeneity was confirmed, and Games–Howell when neither sample size nor homogeneity was confirmed (Shingala et al., 2015) (Tables S4 and S5). The statistical significance level was set to *p* < 0.05.

### 2.2. Study 2. Effect on Body Composition and Physical Fitness by the Degree of Obesity in Children after a 16-Week Exercise Intervention

#### 2.2.1. Design, Participants

Study 2 was conducted to examine the effects of a 16-week exercise program (60-min exercise intervention program three times a week) on the body composition and physical fitness in children by the degree of obesity. A total of 101 children from Study 1 wishing to participate in the exercise intervention program were included. All participants were divided into four groups: NO; *n* = 19, OV; *n* = 15, OB; *n* = 35, and SOB; *n* = 32. All body composition and physical fitness variables were measured before and after the exercise program.

#### 2.2.2. Exercise Program

**Figure 1:**
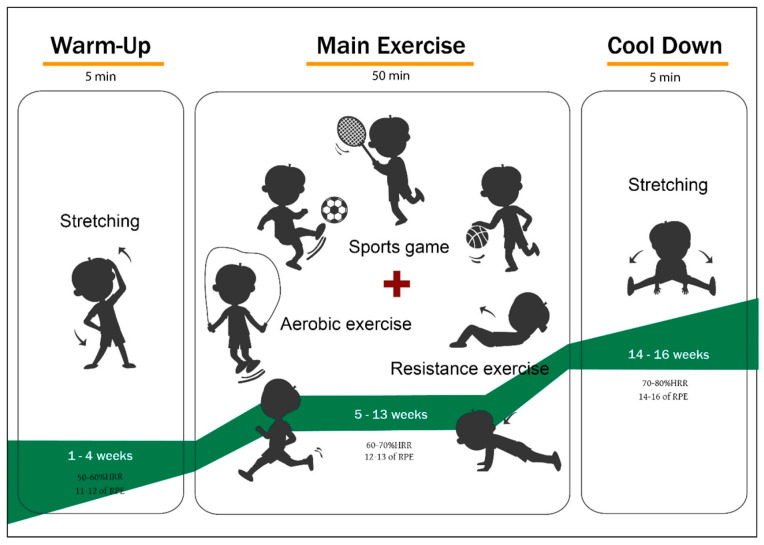
Exercise Program.

The exercise program consisted of individually chosen exercises by each participant and a reinforcement exercise every four weeks. The exercise intensity was 60 min per session (5 min of warm-up, 50 min of the main workout, 5 min of cool-down). It was performed under the supervision of expert guidance at 50–60% heart rate reserve (HRR) (weeks 1–4), 60–70% HRR (weeks 5–13), and 70–80% HRR (weeks 14–16). The exercise program is presented in Figure 1.

#### 2.2.3. Statistical Analyses

The required sample size was calculated using the G-power version 3.1 Window program (Kiel University, Kiel, Germany), based on a 0.25-point effect size (default), an alpha level of 0.05, and over the 95% power. The mean and standard deviation were calculated using (IBM Corp., Chicago, IL, USA). Two-way repeated-measure ANOVA was used to examine the changes between groups and periods after the 16-week exercise program, and Bonferroni was performed for post-hoc testing. The statistical significance level was set to *p* < 0.05.

### 2.3. Study 3. Parameters on Changes in Body Composition and Physical Fitness after Exercise Program (MGPA)

#### 2.3.1. Design

Study 3 is an MGPA to investigate the parameters that affect body composition and physical fitness changes by the degree of obesity after a 16-week exercise program. The data in Study 3 were analyzed, and the pre- and post-difference values based on the measurements in Study 2 were calculated.

#### 2.3.2. Statistical Analyses

**Figure 2:**
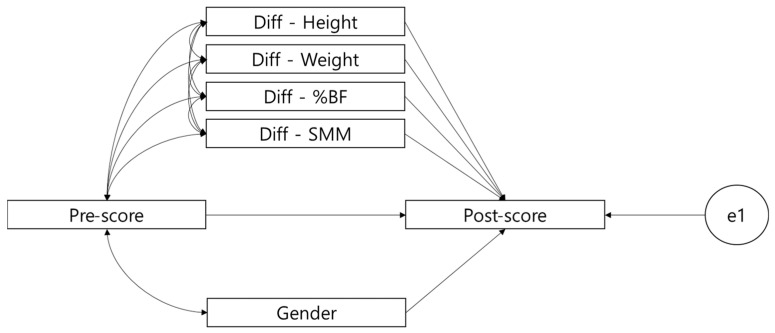
Schematic of the MGPA analysis model.

The statistical significance of the established path was determined by applying the MGPA using the Amos 23.0 Graphics program. Maximum likelihood (ML) was applied to estimate MGPA, and model suitability was determined based on χ^2^, the Tucker–Lewis index (TLI) (>0.9), the comparative fit index (CFI) (>0.9), and the root mean square error of approximation (RMSEA) (<0.08) [19]. All statistical significance levels were set to *p* < 0.05. For setting the MGPA model, the analysis of covariance model (ANCOVA) was applied. In a typical ANCOVA model, the treatment group is dummy-coded (e.g., experimental group = 1, control group = 0) but, in this study, four groups (normal-weight, overweight, obesity, severe obesity) were simultaneously controlled, i.e., configural invariance was maintained to verify the path effect simultaneously, according to the multigroup analysis method of structural equation modeling (SEM) [20]. Therefore, the model in Figure 2 can verify whether pre-scores affect post-scores, and how the changes in height, body weight, %BF, and MSS affect post-scores, while controlling the pre-scores and considering the four groups at the same time. Here, pre-score and covariance were configured to control for the effects of sex (female = 0, male = 1), and whether there was a difference in the means in the post-scores while controlling the pre-scores was confirmed. When configuring the model, the changes in height, body weight, %BF and SMM as well as covariance were not configured for sex; this is because there was no sex difference in the actual changes, and because if all the corresponding covariances were set it became a saturated model restricting the determination of the model fit.

## 3. Results

### 3.1. Study 1

#### 3.1.1. Differences in Body Composition According to the Degree of Obesity

Table 1 presents all the body composition variables showing differences among the groups based on the obesity classification criteria (BMI) in Korean children. Height showed a significant intergroup difference (*F* = 5.897, *p* < 0.001), and the post-hoc test showed that the difference was greater in the SOB group than in the NO group. Weight also showed a significant intergroup difference (*F* = 73.071, *p* < 0.001), and the post-hoc test results showed that the difference was in the order of the NO, OV, OB, and SOB groups. The %BF showed a significant intergroup difference (*F* = 43.318, *p* < 0.001), and the post-hoc test showed that the OB group was higher than the NO or OV group, while the SOB group was the highest. SMM presented a significant intergroup difference (*F* = 8.869, *p* < 0.001), and the post-hoc test showed that OV, OB, and SOB groups were higher than the NO group.

#### 3.1.2. Differences in Physical Fitness According to the Degree of Obesity

When differences in physical fitness were compared according to the classification of obesity in Korean children using BMI, all variables excluding flexibility showed a significant intergroup difference. The results are shown in Table 2. Muscular strength showed a significant intergroup difference (*F* = 3.256, *p* < 0.05), and the post-hoc test displayed that the SOB group was greater than the NO group. Muscular endurance showed a significant intergroup difference (*F* = 3.235, *p* < 0.05), and the post-hoc test displayed that the NO group was higher than the SOB group. Power showed a significant intergroup difference (*F* = 3.331, *p* < 0.05), and the post-hoc test showed that the NO group was higher than the SOB group. Balance showed a significant intergroup difference (*F* = 3.560, *p* < 0.05), and the post-hoc test showed that the NO group was higher than both OB and SOB groups.

#### 3.1.3. Correlation between Body Composition and Physical Fitness by Obesity Degree

We investigated the correlation between body composition and physical fitness according to the obesity degree. The results are presented in Table 3.

Among the physical variables of the NO group, height had a positive correlation with muscular strength (r = 0.522, *p* < 0.01). Weight was found to be positively correlated with muscular strength (r = 0.467, *p* < 0.01) and flexibility (r = 0.458, *p* < 0.01). Moreover, %BF was negatively correlated with power (r = −0.577, *p* < 0.001). The correlation between body composition and physical fitness for the OV group was as follows: height (r = 0.748, *p* < 0.001), weight (r = 0.765, *p* < 0.001), and SMM (r = 0.670, *p* < 0.001) were positively correlated with h muscular strength.

Among the physical variables of the OB group, height was positively correlated with muscular strength (r = 0.688, *p* < 0.001), power (r = 0.285, *p* < 0.05), and balance (r = 0.376, *p* < 0.01). Weight was positively correlated with muscular strength (r = 0.689, *p* < 0.001) and balance (r = 0.350, *p* < 0.01). Moreover, %BF was negatively correlated with power (r = −0.640, *p* < 0.001). SMM was positively correlated with muscular strength (r = 0.273, *p* < 0.05) and power (r = 0.635, *p* < 0.001).

Among the physical variables of the SOB group, height was positively correlated with muscular strength (r = 0.704, *p* < 0.001) and negatively correlated with flexibility (r = −0.307, *p* < 0.05). Weight was positively correlated with muscular strength (r = 0.561, *p* < 0.001). Moreover, %BF was negatively correlated with power (r = −0.604, *p* < 0.001). SMM was positively correlated with power (r = 0.635, *p* < 0.001).

### 3.2. Study 2

#### 3.2.1. Effect of the Exercise Program on Body Composition in Each Group

**Figure 3:**
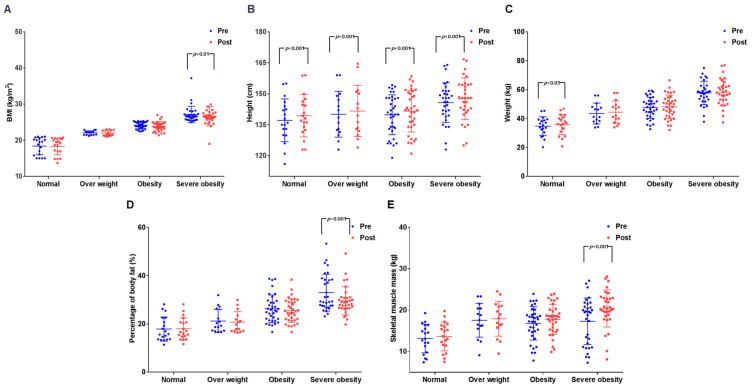
Effects on body composition before and after exercise in each group. (**A**) Change in BMI, significant reduction in the severe obesity group (*p* < 0.01); (**B**) change in height, a significant increase in all groups (*p* < 0.001); (**C**) change in body weight, a significant increase in the normal-weight group (*p* < 0.05); (**D**) change in %BF, significant reduction in the severe obesity group (*p* < 0.001); (**E**) change in SMM, a significant increase in severe obesity group (*p* < 0.001). BMI = body mass index; %BF = percentage of body fat; SMM = skeletal muscle mass.

The results are presented in Figure 3 and Table S4. All body composition variables showed significant differences between the periods and the groups. BMI showed a significant difference between the periods (*p* < 0.05) and among groups (*p* < 0.001), but post-hoc analysis showed that only the SOB group presented a significant difference (*p* < 0.05). Height showed a significant difference among the periods (*p* < 0.001) and the groups (*p* < 0.05), and post-hoc analysis displayed a significant difference among all the groups (*p* < 0.001). Weight showed a significant difference among the periods (*p* < 0.01) and the groups (*p* < 0.001), and post-hoc analysis presented a significant difference only in the NO group (*p* < 0.05). Moreover, %BF showed a significant difference among the periods (*p* < 0.05) and the groups (*p* < 0.001), with a significant interaction effect among periods and groups (*p* < 0.05). Post-hoc analysis showed that a significant difference was present only in the SOB group (*p* < 0.001). Although SMM showed a significant difference among the periods (*p* < 0.001) and the groups (*p* < 0.001) with a significant interaction effect among periods and groups (*p* < 0.05), post-hoc analysis showed that a significant difference was present only in the SOB group (*p* < 0.001). These results demonstrate that exercise affected height in all the groups, and dramatic effects were displayed in the SOB group.

#### 3.2.2. Effects on Physical Fitness of Each Group after the 16-Week Exercise Program

**Figure 4:**
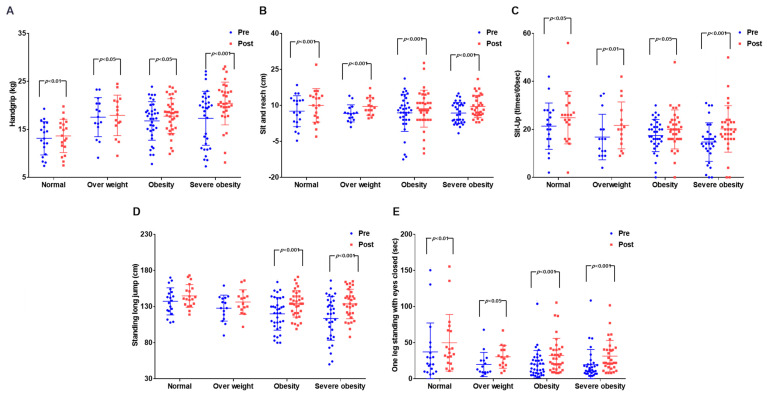
Effect on the physical fitness before and after exercise in each group. (**A**) Change in muscular strength, a significant increase in the severe obesity group (*p* < 0.001); (**B**) change in flexibility, a significant increase in all groups (*p* < 0.001); (**C**) change in muscular endurance, a significant increase in the severe obesity group (*p* < 0.001); (**D**) change in power, a significant increase in the obesity and severe obesity group (*p* < 0.001); (**E**) change in balance, a significant increase in the obesity and severe obesity group (*p* < 0.001).

The results are presented in Figure 4 and Table S5 below. Although significant differences were found between time periods for all physical fitness variables, a significant difference was not found in the interaction between time periods and groups. A significant difference was observed in muscular strength among the time periods (*p* < 0.001) and groups (*p* < 0.05). Post-hoc analysis showed that there was a significant difference in the NO group (*p* < 0.01), OV group (*p* < 0.05), OB group (*p* < 0.05), and SOB group (*p* < 0.001). Flexibility showed a significant difference among time periods (*p* < 0.001), and post-hoc analysis showed that there was a significant difference among all groups (*p* < 0.001). Muscular endurance showed a significant difference among time periods (*p* < 0.001), and post-hoc analysis showed that there was a significant difference in the NO group (*p* < 0.05), OV group (*p* < 0.01), OB group (*p* < 0.05), and SOB group (*p* < 0.001). Agility showed a significant difference among time periods (*p* < 0.001) and groups (*p* < 0.05), and post-hoc analysis showed that there was a significant difference in the OB group (*p* < 0.001) and SOB group (*p* < 0.001). Balance showed a significant difference among time periods (*p* < 0.001) and groups (*p* < 0.05), and post-hoc analysis showed that there was a significant difference in the NO group (*p* < 0.01), OV group (*p* < 0.05), OB group (*p* < 0.001), and SOB group (*p* < 0.001). Post-hoc analysis showed that not all physical fitness variables showed a difference. However, unlike the effects on body composition after the exercise intervention, all of the physical fitness variables of the groups were affected, as the effects of the exercise intervention were the greatest in the SOB group.

### 3.3. Study 3

Table 4 shows the results of applying MGPA in investigating the parameters affecting body composition and physical fitness changes by obesity degree after a 16-week exercise program. When MGPA was applied to pre- and post-scores of muscular strength, results showed χ^2^ = 18.547, Diff = 16, TLI = 0.975, CFI = 0.995, and RMSEA = 0.041. When MGPA was applied to the pre- and post-scores of flexibility, the results were χ^2^ = 18.547, Diff = 16, TLI = 0.968, CFI = 0.994, and RMSEA = 0.041. When MGPA was applied to pre- and post-scores of muscular endurance, the results were χ^2^ = 18.547, Diff = 16, TLI = 0.963, CFI = 0.993, and RMSEA = 0.041. When MGPA was applied to pre- and post-scores of agility, the results were χ^2^ = 18.547, Diff = 16, TLI = 0.968, CFI = 0.994, and RMSEA = 0.041. When MGPA was applied to pre- and post-scores of balance, the results were χ^2^ = 18.547, Diff = 16, TLI = 0.967, CFI = 0.994, and RMSEA = 0.041. Since the pre-score of the physical fitness variables in all four groups had a positive effect on the post-score, it was determined that controlling the pre-score was a reasonable explanation of the individual differences in the post-scores.

#### 3.3.1. MGPA: NO Group

In muscular strength, Diff_Weight was found to have a positive effect on post-hoc muscular strength (*β* = 0.406, *p* < 0.001), while other variables were not statistically significant, meaning the increase in Diff_Weight also increased the post-hoc muscular strength. In flexibility, Diff_SMM was found to have a positive effect on post-hoc flexibility (*β* = 0.213, *p* < 0.05), while other variables were not statistically significant, meaning the increase in Diff_SMM also increased the post-hoc flexibility. In muscular endurance, Diff_%BF was found to have a positive effect on post-hoc muscular endurance (*β* = 0.284, *p* < 0.01), while other variables were not statistically significant, meaning the increase in Diff_%BF also increased the post-hoc muscular endurance. In balance, Diff_Weight (*β* = 0.227, *p* < 0.01), Diff_%BF (*β* = 0.333, *p* < 0.001), and Diff_SMM (*β* = 0.198, *p* < 0.01) were found to have a positive effect on post-hoc balance. Therefore, the increase in Diff_Weight, Diff_%BF, and Diff_SMM also increased the post-hoc balance.

In summary, it was found that body composition variables had the greatest effects on the balance after exercise intervention in the NO group.

#### 3.3.2. MGPA: OV Group

In muscular strength Diff_Height (*β* = 0.291, *p* < 0.001), Diff_SMM (*β* = 0.119, *p* < 0.05), and sex (*β* = 0.084, *p* < 0.05) were found to have a positive effect on post-hoc muscular strength while Diff_%BF had a negative impact on post-hoc muscular strength (*β* = −0.170, *p* < 0.001). Therefore, the increase in Diff_SMM and Diff_Height also increased the post-hoc muscular strength, and the mean post-hoc muscular strength was higher in boys than in girls. However, the rise in Diff_%BF decreased post-hoc muscular strength.

In flexibility, Diff_Height (*β* = 0.497, *p* < 0.05) positively affected post-hoc flexibility, and other variables were not statistically significant. Therefore, the increase in Diff_Height also increased post-hoc flexibility.

In agility, Diff_Height (*β* = 0.215, *p* < 0.01), Diff_Weight (*β* = 0.175, *p* < 0.01), Diff_%BF (*β* = 0.128, *p* < 0.01) and Diff_SMM (*β* = 0.171, *p* < 0.01) had a positive effect on post-hoc agility. Therefore, the increase in Diff_Height, Diff_Weight, Diff_%BF, and Diff_SMM also increased post-hoc agility.

Thus, the physical variables after exercise intervention had the greatest effect on muscular strength and agility in the OV group.

#### 3.3.3. MGPA: OB Group

In flexibility, Diff_Height (*β* = 0.150, *p* < 0.05) positively affected post-hoc flexibility, and other variables did not have a statistically significant effect. Thus, the increase in Diff_Height also increased post-hoc flexibility.

In muscular strength, Diff_Height (*β* = 0.216, *p* < 0.05) and Diff_Weight (*β* = 0.338, *p* < 0.05) had a positive effect on post-hoc muscular strength while Diff_%BF (*β* = −0.909, *p* < 0.05) and Diff_SMM (*β* = −0.875, *p* < 0.05) had a negative effect on post-hoc muscular strength. Therefore, the increase in Diff_Height and Diff_Weight also increased post-hoc muscular strength, but the increase in Diff_%BF and Diff_SMM decreased post-hoc muscular strength.

In balance, Diff_%BF (*β* = 1.269, *p* < 0.05) and Diff_SMM (*β* = 1.426, *p* < 0.05) were found to have a positive effect on post-hoc balance. Therefore, the increase in Diff_%BF and Diff_SMM also increased post-hoc balance.

Hence, the physical variables after exercise intervention were found to have the greatest effect on the OB group’s muscular strength.

#### 3.3.4. MGPA: SOB Group

In muscular strength, Diff_Height had a positive effect on post-hoc muscular strength (*β* = 0.278, *p* < 0.001), and other variables did not have a statistically significant effect. Therefore, the increase in Diff_Height also increased post-hoc muscular strength.

In muscular endurance, Diff_Height (*β* = 0.594, *p* < 0.001) and sex (*β* = 0.275, *p* < 0.05) were found to have a positive effect on post-hoc muscular endurance, and other variables did not have a statistically significant effect. Therefore, boys had a higher mean post-hoc muscular endurance than girls, and the increase in Diff_Height also increased the post-hoc muscular endurance.

In agility, Diff_SMM (*β* = 0.708, *p* < 0.05) and sex (*β* = 0.349, *p* < 0.001) were found to have a positive effect on post-hoc agility. Therefore, the increase in Diff_SMM also increased post-hoc agility while boys had a higher mean post-hoc agility than girls.

## 4. Discussion

We believed that it was necessary to systematically analyze body composition and physical fitness in detail by classifying obesity in children, and thus devised the following three study designs: first, we identified the relationship between body composition and physical fitness according to the degree of obesity classified based on BMI; second, we identified the effects of an exercise intervention on body composition and physical fitness according to the degree of obesity; third, we examined the effects of exercise on body composition and physical fitness according to the degree of obesity. Simultaneously, the following are discussed by verifying the path effects among the groups.

First, this study was conducted with the hypothesis that the severe obesity (SOB) group would be more negative on health in body composition and physical fitness than other groups to identify the relationship between body composition and physical fitness according to the degree of obesity. To this end, a total of 164 ten-year-olds were classified according to the degree of obesity (NO = 33, OV = 34, OB = 54, SOB = 43) to identify the differences in body composition and physical fitness according to the degree of obesity, and to analyze the correlation between body composition and physical fitness. As a result, physical fitness was higher in the SOB group, as we hypothesized, but the results for the rest of the variables excluding physical strength contrasted with the hypothesis. When the correlation between body composition and physical fitness was analyzed according to the degree of obesity, physical strength was associated with height and body weight in all groups, while agility had a negative correlation with %BF in the NO, OB, and SOB groups. The OB and SOB groups showed a positive correlation with SMM. Our study results suggest that body composition and physical fitness are associated according to the obesity degree in children, and that body composition is significantly correlated with physical strength and agility.

Although opinions on BMI remain controversial among researchers, some studies report that height and BMI have a high correlation [21]. An increase of one unit in BMI during childhood leads to a difference of 0.23 cm in boys and 0.29 cm in girls [22]. In other studies that examined multinational samples of 9- to 11-year-olds, a high correlation was found between BMI and total BF index across countries, as BMI was also highly correlated with %BF [23]. Furthermore, in overweight and obese children, there is a higher positive correlation with other variables: fat mass (FM), FM% (FM divided by body weight), FM index (FMI) (FM divided by height squared), and SMM, SM index (SMI) (SMM divided by height squared) compared with normal children [24]. The results of this study show that height, weight, %BF, and SMM show a high correlation (*p* < 0.001) to the physical fitness in the different obesity groups; post-hoc analysis results showed that all body composition variables were higher in the OV group than those in the NO group, supporting the results of the previous research. Higher calorie intake leads to increasing obesity and, in children, adequate nutrition intake increases height, weight, %BF, and SMM. The results of study 1 confirmed that 10-year-old children had a larger physique according to their obesity degree. These results suggest that obese children grow faster. However, rapid growth in childhood can slow growth after puberty [22], and high %BF in childhood has a negative effect on bone health [25]. Thus, obesity at a young age due to excessive nutrient intake or a lack of physical activity should be monitored carefully.

According to a fitness survey conducted by Chen et al. (2002) [26] with 444,652 male students and 433,555 female students, the probability of receiving poor fitness test results was 2.7 times higher in students with BMI > 95th percentile than in those with BMI < 85th percentile, and 1.8 times higher in students with BMI in the 85–94th percentile.

This study found similar results for muscular strength, endurance, and cardiorespiratory endurance when BMI was high, though no significant difference was observed in flexibility. In addition, when flexibility, long jump, sit-ups, and cardiorespiratory endurance were compared with BMI in 102,765 boys aged 9–18, higher BMI was associated with lower results for sit-ups and long-jump distance, while flexibility results varied [27]. In this study, a significant difference was also found in muscular strength, muscular endurance, agility, and balance (*p* < 0.05) but flexibility when the difference in physical fitness was evaluated by the obesity degree. However, SOB was found to be high in muscular strength only, and the results for muscular endurance, agility, and balance contradicted our hypothesis. Agility was higher in the OV group, with significantly lower %BF even though OV and SOB had similar SMM values.

As a result of examining the relationship between anthropometric variables and grip strength for children between the ages of 8 and 11, height was highlighted as the most important variable predicting grip strength in stepwise multiple regression analysis [28]. In addition, as a result of analyzing health-related physical variables in 2474 adolescents, overweight and obese adolescents showed a higher performance in muscular strength and a lower performance in physical fitness related to weight, such as hanging and high jump [29]. Wearing et al. (2006) [30] reported that the grip strength of obese children was relatively higher than that of children of normal weight, but performance tended to be lower with higher weight in tests affected by gravity. Height and weight directly correlate with grip strength [31] and are closely related to height because they are affected by hand size [32].

This study’s results, analyzing the correlation between body composition and physical fitness by obesity degree, show a positive correlation between muscular strength and all body composition variables. Stepwise multiple regression analysis showed a significant effect only in height. However, there was a relationship between height and weight when body composition and physical fitness were analyzed by obesity degree. It was also related to SMM in the OV and OB groups. These results show that greater height is associated with larger hands, and more efficiency in applying a greater force. Muscular strength was shown to be higher in the OV and OB groups with higher SMM than in the NO group.

As for the correlation between body composition and agility, BMI and %BF showed a negative correlation, and SMM showed a positive correlation. In the multiple regression analysis, %BF showed a negative correlation and SMM showed a positive correlation. In the correlation between body composition and agility by obesity degree, there was a negative correlation with %BF in all groups except the OV group. A positive correlation with SMM was found only in the OB and SOB groups. These results indicated that %BF has a negative correlation and SMM has a positive correlation with agility, for which instantaneous explosive power is required.

In the correlation between body composition and flexibility, no significant correlation was found with all body composition variables. However, in the correlation between body composition and flexibility by obesity degree, there was a positive correlation with weight in the NO group and a negative correlation with height in the SOB group. The results of this study support the findings of the study by Huang and Malina (2010), which reported that flexibility varies according to different variables. As sit-and-reach is measured by bending the upper body forward in a sitting position, it seems that a negative correlation was found for the SOB group despite the greatest height (longer legs).

In the correlation between body composition and muscular endurance, BMI and %BF showed a negative correlation. Multiple regression analysis showed a negative correlation with BMI and a positive correlation with body weight. However, a significant difference was not found in any of the groups in the correlation between body composition and muscular strength by obesity degree.

In the correlation between body composition and balance, BMI and SMM showed a negative correlation. Multiple regression analysis showed a significant correlation among SMM, height, and %BF. However, in the correlation between body composition and balance by obesity degree, height and weight were related only in the OB group.

As we hypothesized, the SOB group had a taller physique than other groups, but the results for the rest of the variables except physical fitness were contrary to our hypothesis. When the correlation between body composition and physical fitness was analyzed by obesity degree, muscular strength was related to height and weight in all groups, while agility was negatively correlated with %BF in the NO, OB, and SOB groups, and positively correlated with SMM in the OB and SOB groups. These results seem to suggest that there is a relationship between body composition and physical fitness in children according to obesity degree, and that body composition has a significant correlation with muscular strength and agility. The results of previous studies also showed a similar trend, reporting an inverse correlation—lower physical fitness was seen when body weight or %BF was higher in children [33]. Nevertheless, there were difficulties in identifying the relationship between body composition and physical fitness by obesity degree.

To examine the exercise program effects on body composition and physical fitness by obesity degree under the hypothesis that “the SOB group would have the greatest change in body composition and physical fitness after the 16-week exercise program intervention”, this study divided 101 children who participated in Study 1 and wanted to participate in an exercise intervention program into four groups by obesity degree (NO = 19, OV = 15, OB = 35, SOB = 32) to administer a 16-week exercise intervention program that lasted 60 min per session and was conducted three times a week. As a result, post-hoc analysis showed that the SOB group demonstrated dramatic changes in BMI, %BF, and SMM compared with the other groups, and height showed a significant difference in all groups. When the effects on physical fitness were analyzed by the obesity degree after the exercise program (results of pre- and post-hoc analysis of effects on physical fitness), the SOB group demonstrated dramatic changes in all physical variables in line with the hypothesis of the study compared with other groups, and physical variables except agility showed a significant difference in all groups. These results suggest that exercise program intervention can positively affect the growth and physical fitness in children, particularly in the SOB group.

Recently, Kowal et al. (2021) [34] reported a positive correlation between height and testosterone levels after exercise. Height was not associated with pre-exercise testosterone levels, but taller men had greater post-exercise testosterone levels. Several factors may influence height, for example, a rapid increase in growth hormones during puberty can have a direct effect [35]. These results suggest the possibility that exercise may affect children’s height. Among the effects of the exercise program on body composition, height as a variable was affected in all groups, according to the post-hoc analysis results. An interesting finding in this study was that BMI and %BF were significantly decreased, and SMM was significantly increased only in the SOB group. The reasoning behind these results might be that normal-weight children require a lot of activity to maintain their weight, while obese children can achieve the same effect even with a moderate level of exercise [36]. This study showed a dramatic effect in the SOB group only in terms of body composition after exercise intervention. Thus, a 16-week exercise program intervention can affect the growth in height in children with the greatest effect for the SOB group.

Over the past decades, several studies have been conducted to verify the effects of exercise on physical fitness levels. The effects of exercise were studied with various groups such as children, adolescents, adults, and the elderly, constantly proving exercise to have a positive effect [14,37,38]. Similarly, the findings of this study also showed that conducting an exercise program significantly increased all variables of physical fitness, except for agility, in all groups. Agility was significantly increased only in the OB and SOB groups, with the greatest changes in muscular strength, SMM, and %BF. Another study reported that participants with higher BMI may experience better exercise results than those with lower BMI as they have a higher muscular mass [37]. Thus, it seems that the 16-week exercise program intervention for children had a positive effect, particularly in the SOB group.

This study applied the multi-group path model on the results of Study 2 to examine the effects of exercise on body composition and physical fitness by the obesity degree. As a result, the effects of exercise program intervention on physical fitness were significant in all groups. In this study, Diff_Height, weight, %BF, and SMM can be said to be the variables including growth and exercise intervention effects when considering the characteristics of the subjects. Specifically, according to the results of Study 2, Diff_Height, weight, %BF, and SMM are heavily affected by exercise intervention, and it is very important to interpret the change in physical fitness considering these factors.

It is known that height plays an important role in predicting muscular strength [28] and the results of this study also showed that muscular strength affects height (Table S3). There were significant differences in height and weight in all groups. A significant correlation was found for SMM in the OV and OB groups (correlation between body composition and muscular strength by obesity degree) (Table 3). The results of the MGPA model showed that OV was affected by Diff_Height, %BF, SMM, and sex in terms of effects on muscular strength. This led to sex differences, among which Diff_Height had the greatest effect (Table 4). These results show that the OV group’s muscular strength was most affected by the difference in body composition after exercise intervention.

Muscular endurance was found to negatively correlate with BMI in a study that analyzed upper extremity muscular strength and abdominal muscular endurance by classifying the degree of obesity in percentiles among children aged 10–13 years [39]. In the results of the correlation analysis between body composition and muscular endurance in this study, BMI and %BF showed a negative correlation (Table S2). In contrast, the result of the multiple regression analysis showed that muscular endurance had a negative correlation with BMI, and a positive correlation with weight (Table S3). Furthermore, the results of the MGPA model in investigating the effects of exercise intervention showed a positive correlation for Diff_Height and weight, and a negative correlation for Diff_%BF and SMM, particularly for the OB group (Table 4).

In terms of flexibility, obesity is associated with lower scores in the long jump, as the strength of the lower extremities is decreased [40]. It was reported that overweight and obese adolescents showed lower performance than those with normal weight [29]. Our multiple regression analysis results showed the same results, presenting a negative correlation with %BF and a positive correlation with SMM (Table S3). Analyzing the correlation between body composition and agility by obesity degree, there was a negative correlation with %BF of the NO, OB, and SOB groups, and a positive correlation with SMM of the OB and SOB groups (Table 3). These results are identical to the results of the MGPA model: Diff_SMM of the OV and SOB groups show a positive correlation with agility. However, this is contrary to the finding that a positive correlation exists with the OV group’s Diff_Height, weight, and %BF. We should note that a positive correlation exists between agility and SMM, and it is a well-established theory (reference). However, as children in the OV group are in a pre-obesity stage, the differences in the variables of body composition after exercise intervention can positively affect muscular strength and agility.

According to the survey by Wearing et al. (2006), it was reported that balance is a basic characteristic of motor development. Still, in the case of obese children, as the joint torque value for stabilizing the body changes, the posture may collapse, and it may become difficult to maintain balance. In the results of our correlation analysis of body composition and balance by obesity degree, only the height and weight of OB showed a positive correlation (Table 3). In the result of the MGPA model, Diff_%BF and SMM of OB showed a positive correlation, while Diff_Weight, %BF, and SMM showed a positive correlation in the NO group. These results suggest that the differences in body composition variables after exercise intervention affected balance in children of the NO group.

Our study’s limitation is that the participants were 10-year-old children, and the variables for physical fitness were limited. Therefore, it would be difficult to generalize the findings to all age groups. There were difficulties in predicting the outcomes for physical fitness variables other than grip strength, sit-and-reach, sit-ups, long jump in place, and standing on one leg. Further research is needed to analyze other variables of physical fitness within different age groups. In addition, although the effect size was calculated using the G-power program, there is a limitation that the sample size is not sufficient. Although the results of this study are insufficient to make a complete generalization, it will nevertheless be possible to present the overall body composition and physical fitness flow of children according to their degree of obesity.

## 5. Conclusions

Overall, this is the first study analyzing the relationship between body composition and physical fitness in children, according to obesity degree. The results showed that the larges values for all variables of body composition were in the SOB group. Muscular strength was also the highest in the SOB group, and balance was the highest in the NO group. Agility was the highest in the OV group. When the correlation between body composition and physical fitness was analyzed by the obesity degree, muscular strength was associated with height and body weight in all groups. In contrast, agility negatively correlated with %BF in the NO, OB, and SOB groups. The OB and SOB groups showed a positive correlation with SMM. Second, when the effects of the 16-week exercise program intervention on body composition and physical fitness were analyzed according to the obesity degree, significant differences were only found in the SOB group for BMI, %BF, and SMM in terms of effects on body composition, while height showed a significant difference in all groups. In terms of the effects of physical strength, all groups showed a significant increase in muscular strength, flexibility, muscular endurance, and balance, with the greatest increase observed for the SOB group. Agility showed a significant increase only in the OB and SOB groups. It seems that exercise could help children grow in height, with the SOB group benefiting the most from exercise. Third, after a 16-week exercise program intervention, the greatest number of physical variables affected balance, muscular strength and agility, and muscular endurance in the NO, OV, and OB, respectively. Thus, the SOB group had a negative on health in body composition than the other groups, while physical fitness varied depending on the obesity degree. These results confirm, with clinical significance, that it is important to configure an appropriate exercise program according to the degree of obesity, to effectively prevent and improve childhood obesity.

## Figures and Tables

**Table 1:** Differences in body composition by obesity degree.

	Group	NO (*n* = 33)	OW (*n* = 34)	OB (*n* = 54)	SOB (*n* = 43)	*F*	Post-hoc
Variable							
BMI (kg/m^2^)		18.50 ± 1.98	21.94 ± 0.55	24.14 ± 0.86	27.20 ± 2.31	202.664 ***	NO < OW < OB < SOB (Games-Howell)
Height (cm)		135.52 ± 9.92	138.68 ± 10.22	140.35 ± 10.09	144.77 ± 8.94	5.897 ***	NO < SOB (Scheffe)
Weight (kg)		34.09 ± 5.60	42.45 ± 6.52	47.86 ± 7.36	57.14 ± 7.66	73.071 ***	NO < OW < OB < SOB (Scheffe)
%BF (%)		17.94 ± 4.75	21.27 ± 4.29	26.53 ± 5.90	31.97 ± 7.17	43.318 ***	NO, OW < OB < SOB (Games-Howell)
SMM (kg)		13.26 ± 2.83	17.02 ± 3.31	16.81 ± 4.22	17.87 ± 5.06	8.869 ***	NO < OW, OB, SOB (Games-Howell)

Overweight: BMI 85th–94th percentile (Girl: 20.6~22.39, Boy: 21.2~23.09); Obesity: BMI 95th–98th percentile (Girl: 22.4~24.79, Boy: 23.1~25.4); Severe obesity: BMI ≥ 99th percentile (Girl: ≥24.8, Boy: ≥25.5); NO: Normal; OW: Overweight; OB: Obesity; SOB: severe obesity. Note: *** *p* < 0.001.

**Table 2:** Differences in physical fitness by obesity degree.

	Group	NO (*n* = 33)	OW (*n* = 34)	OB (*n* = 54)	SOB (*n* = 43)	*F*	Post-hoc
Variable							
Muscular strength (kg)		13.95 ± 3.54	16.01 ± 4.10	16.09 ± 4.40	17.02 ± 4.86	3.256 *	NO < SOB (Scheffe)
Flexibility (cm)		7.26 ± 6.00	6.86 ± 4.17	6.87 ± 7.71	7.04 ± 4.69	0.035	
Muscular endurance (times)		20.45 ± 10.23	18.32 ± 10.17	17.39 ± 8.36	13.98 ± 8.96	3.235 *	SOB < NO (Scheffe)
Power (cm)		123.65 ± 26.76	128.18 ± 16.83	119.43 ± 26.16	110.36 ± 30.45	3.331 *	SOB < OW (Games-Howell)
Balance (s)		38.96 ± 49.73	24.32 ± 18.05	20.33 ± 18.27	21.05 ± 19.88	3.560 *	OB, SOB < NO (Games-Howell)

Overweight: BMI 85th–94th percentile (Girl: 20.6~22.39, Boy: 21.2~23.09); Obesity: BMI 95th–98th percentile (Girl: 22.4~24.79, Boy: 23.1~25.4); Severe obesity: BMI ≥ 99th percentile (Girl: ≥24.8, Boy: ≥25.5); NO: Normal; OW: Overweight; OB: Obesity; SOB: severe obesity. Note: * *p* < 0.05.

**Table 3:** Correlation between body composition and physical fitness by obesity degree.

Variable	Muscular Strength	Flexibility	Muscular Endurance	Power	Balance
**NO**					
BMI	0.005	−0.011	−0.292	−0.273	−0.037
Height	0.522 **	−0.235	0.162	−0.042	0.232
Weight	0.467 **	0.458 **	−0.045	−0.225	0.212
%BF	0.073	0.038	−0.224	−0.577 ***	0.252
SMM	0.097	−0.177	−0.070	0.182	−0.337
**OW**					
BMI	0.294	−0.203	0.001	0.196	0.063
Height	0.748 ***	0.104	0.119	0.331	0.188
Weight	0.765 ***	0.084	0.106	0.323	0.173
%BF	0.078	0.274	−0.144	−0.223	−0.141
SMM	0.670 ***	−0.005	0.101	0.336	0.209
**OB**					
BMI	0.227	−0.268	−0.063	−0.027	−0.017
Height	0.688 ***	−0.067	0.251	0.285 *	0.376 **
Weight	0.689 ***	−0.127	0.227	0.258	0.350 **
%BF	−0.022	0.046	−0.004	−0.640 ***	0.113
SMM	0.273 *	−0.222	0.052	0.635 ***	−0.052
**SOB**					
BMI	−0.134	0.019	−0.104	−0.255	−0.064
Height	0.704 ***	−0.307 *	0.279	0.239	0.055
Weight	0.561 ***	−0.250	0.202	0.064	0.014
%BF	0.103	−0.057	−0.197	−0.604 ***	0.193
SMM	−0.056	0.003	0.243	0.635 ***	−0.288

NO = Normal; OW = Overweight; OB= Obesity; SOB = Severe obesity. Note: * *p* < 0.05, ** *p* < 0.01, *** *p* < 0.001.

**Table 4:** A multi-group path analysis (MGPA) model result.

Group	Variables	Muscular Strength			Flexibility			Muscular Endurance			Power			Balance		
		*b*	*β*	*t*	*b*	*β*	*t*	*b*	*β*	*t*	*b*	*β*	*t*	*b*	*β*	*t*
**NO**	**Pre_F**	0.956	0.935	8.289 ***	0.921	0.861	11.328 ***	1.106	0.971	11.117 ***	0.795	0.942	11.941 ***	0.834	0.887	8.012 ***
	**Diff_** **Height**	−0.120	−0.047	−0.687	0.339	0.086	1.139	0.789	0.130	1.322	−0.113	−0.013	−0.160	−1.232	−0.061	−0.935
	**Diff_** **Weight**	1.143	0.406	4.025 ***	−0.447	−0.102	−1.062	1.065	0.158	1.309	0.265	0.027	0.267	5.074	0.227	2.719 **
	**Diff_** **%BF**	−0.341	−0.081	−0.973	0.359	0.055	0.769	2.862	0.284	3.130 **	1.241	0.085	0.959	11.134	0.333	3.981 ***
	**Diff_** **SMM**	−1.611	−0.186	−1.219	2.863	0.213	2.084 *	2.928	0.141	1.190	4.756	0.159	1.627	13.681	0.198	2.819 **
	**Sex**	−0.531	−0.059	−0.896	−0.382	−0.027	−0.443	1.815	0.085	1.099	1.710	0.055	0.856	−6.052	−0.085	−1.738
**OW**	**Pre_F**	0.772	0.614	10.082 ***	1.151	1.271	2.979 **	0.675	0.665	3.309 ***	0.829	0.921	16.121 ***	0.794	0.836	5.429 ***
	**Diff_** **Height**	0.772	0.291	4.150 ***	1.286	0.497	1.972 *	1.334	0.220	0.683	2.326	0.215	2.885 **	1.100	0.111	0.449
	**Diff_** **Weight**	−0.290	−0.058	−1.199	1.143	0.233	0.798	0.684	0.060	0.228	3.567	0.175	3.040 **	0.945	0.050	0.247
	**Diff_** **%BF**	−0.446	−0.170	−3.316 ***	1.385	0.541	1.689	−1.547	−0.258	−1.039	1.373	0.128	2.217 *	−2.678	−0.273	−1.458
	**Diff_** **SMM**	1.629	0.119	2.475*	−2.386	−0.178	−0.857	−4.852	−0.155	−0.555	9.589	0.171	2.729 **	−2.928	−0.057	−0.293
	**Sex**	0.769	0.084	2.294 *	4.627	0.517	1.428	2.042	0.098	0.531	−0.377	−0.010	−0.214	−5.257	−0.153	−1.087
**OB**	**Pre_F**	1.051	0.839	12.173 ***	0.886	0.893	14.562 ***	0.924	0.756	9.213 ***	0.669	0.852	6.767 ***	0.902	0.713	5.362 ***
	**Diff_** **Height**	0.391	0.096	1.200	0.963	0.150	2.141 *	1.445	0.216	2.093 *	1.687	0.113	0.819	2.507	0.127	0.872
	**Diff_** **Weight**	−0.230	−0.084	−0.809	−0.049	−0.011	−0.125	1.515	0.338	2.510 *	0.005	0.000	0.003	−3.563	−0.270	−1.334
	**Diff_** **%BF**	0.521	0.376	1.361	−0.449	−0.205	−0.845	−2.075	−0.909	−2.514 *	−0.063	−0.012	−0.026	8.527	1.269	2.186 *
	**Diff_** **SMM**	0.909	0.487	1.683	−0.382	−0.129	−0.506	−2.692	−0.875	−2.299 *	3.058	0.447	0.892	12.923	1.426	2.396 *
	**Sex**	0.783	0.076	1.234	−0.361	−0.022	−0.373	−1.711	−0.101	−1.266	0.058	0.002	0.014	−6.916	−0.139	−1.228
**SOB**	**Pre_F**	0.826	0.691	8.352 ***	0.824	0.794	7.035 ***	0.622	0.548	3.821 ***	0.460	0.637	5.900 ***	0.405	0.395	3.151 **
	**Diff_** **Height**	0.787	0.278	3.471 ***	0.123	0.059	0.334	2.759	0.594	3.414 ***	−1.168	−0.114	−0.807	2.735	0.273	1.610
	**Diff_** **Weight**	−0.067	−0.035	−0.430	−0.023	−0.016	−0.093	−0.684	−0.218	−1.264	−1.292	−0.187	−1.312	−0.180	−0.027	−0.157
	**Diff_** **%BF**	−0.051	−0.052	−0.299	0.006	0.009	0.023	0.622	0.388	0.947	−0.326	−0.092	−0.297	0.872	0.252	0.701
	**Diff_** **SMM**	0.092	0.077	0.493	0.124	0.141	0.425	0.436	0.223	0.628	3.050	0.708	2.544 *	2.360	0.560	1.798
	**Sex**	1.505	0.115	1.710	−1.430	−0.149	−1.406	5.884	0.275	2.139 *	16.446	0.349	3.830 ***	3.484	0.076	0.764

NO = Normal; OW = Overweight; OB = Obesity; SOB = Severe obesity; SMM: Skeletal muscle mass. Note: * *p* < 0.05, ** *p* < 0.01, *** *p* < 0.001.

## Data Availability

The authors declare that all data and materials are available to be shared on a formal request.

## Supplementary Materials

The following are available online at https://www.mdpi.com/article/10.3390/ijerph19010487/s1, Table S1: Correlation between BMI, Height, Weight, %BF and SMM according to obesity status, Table S2: Correlation between body composition and physical fitness, Table S3: Effects of body composition on physical fitness: stepwise multiple regression analysis results, Table S4: Effect of exercise program on body composition by obesity in children, Table S5: Effect of exercise program on physical fitness by obesity in children.
