# Supplementary material for: The Relationship between Body Composition and Physical Fitness and the Effect of Exercise According to the Level of Childhood Obesity Using the MGPA Model
Source: Int J Environ Res Public Health. 2022 Jan 2;19(1):487. doi: 10.3390/ijerph19010487 (PMC8744593; doi:10.3390/ijerph19010487)
Supplement: Supplementary file 1 [file ijerph-19-00487-s001.zip › ijerph-1510809-supplementary.pdf]

Table S1. Correlation between BMI, Height, Weight, %BF and SMM according to obesity status

Variable	BMI	Height	Weight	%BF	SMM
**NO**					
BMI	1	-.177	.812**	.192	.496*
Height		1	.419	-.316	.318
Weight			1	-.020	.687**
%BF				1	-.300
SMM					1
**OW**					
BMI	1	-.580*	.233	.194	-.396
Height		1	.650**	-.612*	.696**
Weight			1	-.608*	.473
%BF				1	-.418
SMM					1
**OB**					
BMI	1	-.083	.852**	.211	.024
Height		1	.418*	-.392$^{*.}$	.542**
Weight			1	.059	.234
%BF				1	-.931**
SMM					1
**SOB**					
BMI	1	-.332	.840**	.533**	-.185
Height		1	.227	-.703**	.678**
Weight			1	.148	.200
%BF				1	-.883**
SMM					1

NO=Normal, OW=Overweight, OB= Obesity, SOB=Severe obesity. Note: $^{*}p<.05$, $^{**}p<.01$.

Table S2. Correlation between body composition and physical fitness

Variable	Muscle strength	Flexibility	Muscle endurance	Power	Balance
BMI	.206**	-.042	-.265***	-.256***	-.206**
Height	.687***	-.115	.114	.122	.125
Weight	.570***	-.095	-.090	-.081	-.053
%BF	.180*	.023	-.248**	-.556***	-.039
SMM	.254**	-.120	.014	.430***	-.215**

%BF: Percentage of body fat, SMM: Skeletal muscle mass. Note: *p<.05, **p<.01, ***p<.001.

Pearson's correlation is analyzed to investigate the relationship between body composition variables and physical fitness. Muscle strength is found to be positively correlated with all variables including BMI (r=.206, p<.01), height (r=.687, p<.001), weight (r=.570, p<.001), %BF (r=.180, p<.05) and skeletal muscle mass (r=.254, p<.01). Flexibility does not show a significant correlation with any of the variables. Muscle endurance is found to have a negative correlation with variables BMI (r=-.265, p<.001) and %BF (r=-.248, p<.01). Agility showed a strong correlation with BMI (r=-.256, p<.001), %BF (r=-.556, p<.001) and skeletal muscle mass (r=.430, p<.001). Balance showed a negative correlation with BMI (r=-.206, p<.001) and skeletal muscle mass (r=-.215, p<.01).

Table S3. Effects of body composition on physical fitness: stepwise multiple regression analysis results

	**dependent variable**	***B***	***SE***	***β***	***t***	***DF***	***F***
Height	Muscle strength (Handgrip)	.296	.025	.687	12.038***	1	144.923***
R=.687, R^2=.472, Adjusted R^2=.469							
BMI	Muscle endurance	-1.454	.339	-.525	-4.288***	2	9.925***
Weight		.293	.110	.328	2.675**		
R= .331, R^2=.110, Adjusted R^2= .099							
%BF	Power	-1.655	.215	-.481	-7.685***	2	54.469***
SMM		1.924	.381	.316	5.054***		
R= .635, R^2=.404, Adjusted R^2= .396							
SMM	Balance	-3.091	.595	-.471	-5.197***	3	10.288***
Height		1.135	.251	.407	4.516***		
%BF		-.875	.299	-.236	-2.926**		
R= .402, R^2=.162, Adjusted R^2= .146							

%BF: Percentage of body fat, SMM: Skeletal muscle mass. Note: *p<.05, **p<.01, ***p<.001.

Height predicts grip strength with 46.9% (Adjusted R^2=.469) explanatory power, while height was the only variable among body composition variables that had an effect of .687 on grip strength. BMI and weight were able to predict muscle endurance with 9.9% (Adjusted R^2=.099) explanatory power, and BMI (β=-.525) was found to have a greater effect than weight (β=.328). %BF and SMM predict agility with 63.5% (Adjusted R^2=.635) explanatory power, and %BF (β=-.481) was found to have a greater effect than SMM (β=.316). Height predicts balance with 14.6% (Adjusted R^2=.146) explanatory power, and SMM (β=-.471) was found to have a greater effect than height (β=.407) or %BF (β=-.236).

Table S4. Effect of exercise program on body composition by obesity in children

Variable		NO (n=19)	OW (n=15)	OB (n=35)	SOB (n=32)	2RM		*F*	Post-hoc
BMI	Pret	18.38±2.73	22.05±0.57	24.14±0.92	27.12±2.28	T	4.537*	105.461***	NO<OW<OB<SOB
	Post	18.30±2.33	22.01±0.73	23.85±1.29	26.67±2.65$^{\$\$}$	G	93.970***	73.639***	NO<OW<OB<SOB
	Diff	-0.09±0.71	-0.04±0.40	-0.29±0.81	-0.45±1.39	T×G	.924	.838	
Height	Pre	137.15±10.37	140.10±11.10	139.83±9.69	145.74±9.60	T	104.523***	3.510*	NO<OW<OB<SOB
	Post	139.42±10.28$^{\$\$\$}$	141.66±12.42$^{\$\$\$}$	141.60±10.11$^{\$\$\$}$	147.63±10.13$^{\$\$\$}$	G	3.310*	3.092*	NO<OW<OB<SOB
	Diff	2.26±1.81	1.56±1.58	1.77±1.22	1.89±2.17	T×G	.530	.530	
Weight	Pret	34.75±6.53	43.55±7.22	47.52±7.33	57.78±8.12	T	10.987**	40.335***	NO<OW,OB<SOB
	Post	35.80±6.98	44.46±7.82	48.19±8.08	58.30±9.04	G	36.448***	31.860***	NO<OW,OB<SOB
	Diff	1.05±1.63	0.91±0.84	0.67±1.82	0.53±3.22	T×G	.256	.256	
%BF	Pre	17.89±5.02	21.23±4.82	26.27±5.71	32.98±7.60	T	6.426*	27.699***	NO<OB<SOB
	Post	17.99±4.54	20.76±4.37	25.53±4.83	29.58±5.80$^{\$\$\$}$	G	30.060***	24.468***	NO,OW<OB,SOB
	Diff	0.11±1.09	-0.47±1.60	-0.74±3.58	-3.40±6.27	T×G	3.750*	3.750*	SOB<NO
SMM	Pre	13.14±3.47	17.53±4.05	16.77±4.01	17.31±5.61	T	12.314***	4.143**	NO<OB,SOB
	Post	13.64±3.47	17.92±4.20	17.75±3.64	20.40±4.45$^{\$\$\$}$	G	7.992***	11.510***	NO<OW,OB,SOB
	Diff	0.50±0.53	0.30±0.73	0.98±2.65	3.09±5.15	T×G	3.864*	3.857*	NO,OW<SOB

NO=Normal, OW=Overweight, OB= Obesity, SOB=Severe obesity, %BF: Percentage of body fat, SMM: Skeletal muscle mass.
Note: $^{*}p<.05$, $^{**}p<.01$, $^{***}p<.001$, $^{\$\$}p<.01$. $^{\$\$\$}p<.001$ vs pre-exercise.

Table S5. Effect of exercise program on physical fitness by obesity in children

Variable		NO (n=19)	OW (n=15)	OB (n=35)	SOB (n=32)	2RM		*F*
Muscle strength (kg)	Pre	14.02±4.04	15.71±3.43	15.85±3.99	17.48±4.80	T	36.294***	2.773*
	Post	15.38±4.22$^{\$\$}$	17.01±4.44$^{\$}$	16.73±5.03$^{\$}$	19.55±5.87$^{\$\$\$}$	G	3.054*	3.111*
	Diff	1.37±1.85	1.30±1.73	0.89±2.15	2.07±2.61	T×G	1.626	1.626
Flexibility (times)	Pre	7.59±6.47	6.69±3.56	6.90±7.75	6.82±4.43	T	66.505***	.086
	Post	10.02±6.96$^{\$\$\$}$	9.57±3.65$^{\$\$\$}$	8.65±7.69$^{\$\$\$}$	9.55±4.65$^{\$\$\$}$	G	.909	.239
	Diff	2.42±2.20	2.89±3.13	1.75±2.92	2.73±2.93	T×G	.122	.909
Muscle endurance (times)	Pre	21.37±9.66	16.80±9.49	17.43±6.68	14.72±8.04	T	33.673***	2.659
	Post	24.89±10.90$^{\$}$	21.67±9.77$^{\$\$}$	20.00±8.15$^{\$}$	20.19±9.69$^{\$\$\$}$	G	1.998	1.293
	Diff	3.53±5.32	4.87±8.60	2.57±4.61	5.47±8.18	T×G	1.161	1.161
Power (cm)	Pre	137.26±18.71	127.87±17.92	120.00±22.62	113.59±30.15	T	34.435***	4.205**
	Post	144.68±15.52	136.27±16.82	133.83±18.26$^{\$\$\$}$	133.61±20.36$^{\$\$\$}$	G	3.671*	1.771
	Diff	7.42±6.39	8.40±8.03	13.83±18.09	20.02±28.97	T×G	2.039	2.039
Balance (sec)	Pre	37.21±40.15	19.85±16.75	20.24±18.96	19.55±21.12	T	39.316***	2.538
	Post	49.91±39.30$^{\$\$}$	30.39±15.72$^{\$}$	33.29±23.78$^{\$\$\$}$	31.40±21.81$^{\$\$\$}$	G	2.916*	2.584
	Diff	12.70±20.64	10.54±10.77	12.05±19.23	11.85±16.81	T×G	.043	.043

NO=Normal, OW=Overweight, OB= Obesity, SOB=Severe obesity. Note: $^{*}p<.05$, $^{**}p<.01$, $^{***}p<.001$. $^{\$}p<.05$, $^{\$\$}p<.01$, $^{\$\$\$}p<.001$ vs pre-exercise.
